# Ameliorating effects of bortezomib, a proteasome inhibitor, on development of dextran sulfate sodium-induced murine colitis

**DOI:** 10.3164/jcbn.18-42

**Published:** 2018-06-08

**Authors:** Shigeki Sakai, Atsushi Nishida, Masashi Ohno, Osamu Inatomi, Shigeki Bamba, Mitsushige Sugimoto, Masahiro Kawahara, Akira Andoh

**Affiliations:** 1 Department of Medicine, Shiga University of Medical Science, Seta-Tsukinowa, Otsu 520-2192, Japan

**Keywords:** IBD, proteasome inhibitor, NF-κB

## Abstract

We examined the effect of bortezomib, a proteasome inhibitor, on the development of dextran sulfate sodium (DSS)-induced colitis in mice. DSS-colitis was induced by the administration of 3% DSS in water in C57BL/6J mice. Bortezomib was intraperitoneally administered daily for 9 days from the start of DSS. Ubiquitination of IκBα was evaluated by immunoblot. Bortezomib significantly ameliorated DSS-induced body weight loss and reduced the disease activity. The translocation of NF-κBp65 into the nucleus was markedly suppressed in the DSS + bortezomib group compared to the DSS group, but this difference was not detected in submucosal tissue. Ubiquitinated IκBα in the cytoplasm of colon epithelial cells was increased in the DSS + bortezomib group compared to the DSS group. In HT-29 cells, bortezomib blocked tumor necrosis factor-α (TNF-α)-induced nuclear translocation of NF-κB and this was accompanied by an increase in ubiquitinated IκBα in the cytoplasm. The mRNA expression of inflammatory mediators in colonic epithelial cells was significantly reduced by the treatment of bortezomib. Bortezomib inhibited the nuclear translocation of NF-κB in colonic epithelial cells by suppressing the degradation of IκBα and contributed to an improvement in DSS colitis. Our study suggests that bortezomib may be a new treatment option for IBD.

## Introduction

Inflammatory bowel disease (IBD), Crohn’s disease and ulcerative colitis, is characterized by chronic inflammation of the intestinal tract which undergoes repeated periods of relapse and remission.^(1,2)^ Although the obvious etiology of IBD remains unknown, previous studies have shown that IBD is caused by an excessive immune response to intestinal bacteria and dietary antigens in the intestinal tract.^(3–6)^

The transcription factor NF-κB is composed of five family molecules p65 (RelA), RelB, c-Rel, NF-κB1 (p50/p105), NF-κB2 (p52/p100).^(7)^ NF-κB forms homo- or heterodimers via the Rel homology domain (RHD) on the N-terminal side and binds to DNA. In steady state, an inhibitor of the NF-κB (IκBα) family is bound to RHD of NF-κB via ankyrin repeat, and nuclear localization signals within RHD are masked, keeping localization of NF-κB in the cytoplasm.^(8,9)^ When cells are stimulated by inflammatory stimuli such as tumor necrosis factor-α (TNF-α), interleukin-1β (IL-1β) and toll-like receptors, IκB kinase is activated and phosphorylation of IκBα is induced. Subsequently, ubiquitination of IκBα is induced and ubiquitinated IκBα is degraded by proteasome, leading to translocation of NF-κB into the nucleus.^(9–11)^ NF-κB has various physiological functions to maintain homeostasis of the body, and its abnormal control plays a crucial role in various inflammatory disorders and malignant diseases.^(10,11)^ In IBD, activation of NF-κB is enhanced, and along with this, excessive expression of inflammatory cytokines such as IL-1β, TNFα and IL-6 in the mucosa has been reported.^(12,13)^

Bortezomib is a boronic acid derivative and is a proteasome inhibitor used as a therapeutic agent for hematological diseases such as multiple myeloma. Proteasome is a proteolytic enzyme complex that selectively degrades ubiquitinated proteins and is composed of 20S and 19S proteasomes.^(14)^ The 20S proteasome is composed of proteins each having seven subunits called α rings and β rings. Bortezomib suppresses the function of the β5 subunit-located chymotrypsin-like activity and the β1 subunit-located caspase-like activity, and suppresses the degradation of ubiquitinated protein by proteasome.^(15)^ This proteasome-ubiquitin-modified system plays an important role in the activation of NF-κB, and bortezomib inhibits the degradation of ubiquitinated IκBα by inhibiting the function of proteasome, and as a result, the translocation of NF-κB into the nucleus is blocked.^(16)^ Proteasome inhibitors are thought to be effective treatment options for inflammatory diseases in which abnormal activation of NF-κB is involved in the pathophysiology.

In this study, the effect of bortezomib on IBD was examined using the DSS-induced colitis model.

## Materials and Methods

### Induction of experimental animals and DSS enteritis

C57BL/6J mice (6–8 weeks of age) were purchased from CLEA Japan Inc. (Tokyo, Japan), and kept under a specific pathogen-free environment. Dextran sodium sulfate (DSS) colitis was induced by freely available drinking water containing 3% DSS (molecular weight 36,000–50,000 Da; MP Biomedicals, Santa Ana, CA). The amount of drinking water was measured every day, and the amount of DSS ingested by each mouse was calculated. Mice were divided into 4 groups consisting of a control group, bortezomib group (BTZ), DSS group and DSS + bortezomib group. Bortezomib (Calbiochem, San Diego, CA) (0.35 mg/kg/day) or physiological saline was intraperitoneally administered daily for 9 days from the start of DSS. All experiments were strictly conducted under the guidelines for the care and use of laboratory animals of the National Institutes of Health. This research has been approved by the Shiga University of Medicine Animal Life Science Research Center (permission number: 2016-8-6).

### Evaluation of DSS colitis

The severity of DSS colitis was assessed using the disease activity index (DAI) based on previous reports.^(17)^ Hematoxylin-eosin (HE) staining of formalin-fixed tissues was performed, and histological evaluation of colitis was performed based on the methodology previously reported.^(18)^

### Immunohistochemical staining of NF-κB and Ki-67

 Immunohistochemical staining was performed as previously reported.^(19)^ To evaluate Ki-67 positive cells, the ratio of Ki-67 positive cells to total number of cells in one crypt was calculated. Antibodies used are listed in Supplemental Table 1*.

### Cell culture

The human colonic epithelial cell line HT-29 was purchased from the American Type Culture Collection (ATCC, Manassas, VA). Cells were cultured in Dulbecco’s Eagle’s medium (DMEM) (Nacalai Tesque Inc., Kyoto, Japan) containing 50U/ml penicillin, 50 µg/ml streptomycin and 10% fetal bovine serum. Cells were cultured in 6-well plates at 37°C, 5% CO_2_ for 48 h. Cells were stimulated with 100 ng/ml TNF-α (R&D Systems Inc., Minneapolis, MI) in the presence or absence of bortezomib.

### Isolation of intestinal epithelial cells

Colon epithelial cells were isolated as previously reported.^(20)^ Briefly, the intestines were inverted and transferred to a tube containing 5 ml HBSS, 5 ml EDTA, and 1 mM dithiothreitol. The tube was shaken at 37°C for 20 min. The supernatant was centrifuged and the pellet was resuspended in 40% Percoll and centrifuged. Upper layer cells were collected and used as colon epithelial cells.

### Real-time PCR analysis

Total RNA was extracted using TRIzol (Invitrogen, Carlsbad, CA). Real-time PCR was performed using a LightCycler 480 system (Roche Applied Science, Penzberg, Germany) and SYBR Premix Ex Taq II (TAKARA, Otsu, Japan). Expression of each target molecule was standardized to β-actin and expressed as a relative ratio compared with the control group. The PCR primers used are listed in Supplemental Table 2*.

### Extraction of nuclear and cytoplasmic proteins and immunoblot analysis

Nuclear and cytoplasmic proteins were extracted according to previous reports.^(21)^ Nuclear and cytoplasmic proteins of the isolated epithelial cells were extracted using a CelLytic NuCLEAR Extraction Kit (Sigma-Aldrich Co., St. Louis, MO). The extracted nuclear protein was used for immunoblot against NF-κBp65. Signal detection was performed using a chemiluminescence immunoblot system (GE Healthcare UK Ltd., Little Chalfont, UK). Antibodies used are listed in Supplemental Table 1*.

### Immunoprecipitation analysis

HT-29 cells were stimulated for 12 h with TNF-α (100 ng/ml) in the presence or absence of bortezomib, and then the cytoplasmic protein was extracted using a CelLytic NuCLEAR Extraction Kit (Sigma-Aldrich Co.). The cytoplasmic protein obtained from tissue and HT-29 cells was incubated with anti-IκBα antibody overnight at 4°C, and then incubated with Protein A/G agarose beads (Santa Cruz, Dallas, TX) at 4°C for 1 h. After heating at 95°C for 5 min, immunoblotting was performed on the eluted immunoprecipitate. The antibody used is listed in Supplemental Table 1*.

### Statistical analysis

Data are presented as mean ± SEM. A one-way ANOVA using Bonferroni post hoc tests was used for statistical analysis and a *p* value <0.05 was considered significant.

## Results

### Bortezomib suppressed the development of DSS enteritis

**Fig. 1:**
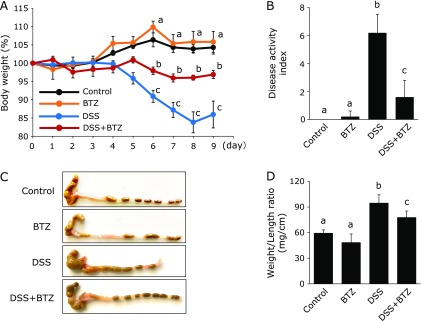
The effect of bortezomib on DSS colitis. (A) Body weight, (B) disease activity index, (C) representative photographs of the colon, and (D) colonic weight/length on day 9. All data are means ± SEM (*n* = 15/group). Values not sharing a letter denote significant differences (*p*<0.05). BTZ, bortezomib.

**Fig. 2:**
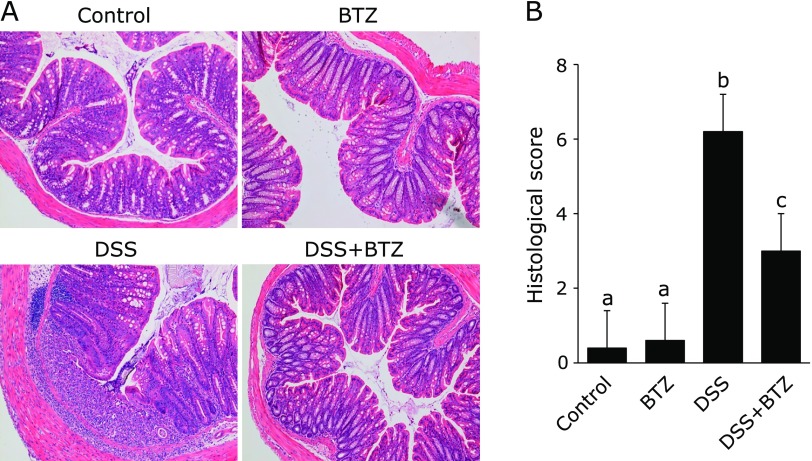
Histological evaluation of disease activity. (A) Picture on day 9 (original magnification ×100) and (B) histological score. All data are means ± SEM (*n* = 10/group). Values not sharing a letter denote significant differences (*p*<0.05). BTZ, bortezomib.

DSS colitis was induced by 3% DSS in water for 5 days. Intraperitoneal administration of physiological saline or bortezomib was performed every 24 h for 9 consecutive days. The severity of colitis was assessed using body weight change and DAI score. As shown in Fig. 1A, weight loss was significantly suppressed in the DSS + bortezomib group but not in the DSS group. The DAI was significantly lower in the DSS + bortezomib group than the DSS group (Fig. 1B). The intestinal tract length was significantly longer in the DSS + bortezomib group than in the DSS group (Fig. 1C). The ratio of weight to length, which is an index of intestinal tissue edema, was significantly lower in the DSS + bortezomib group than in the DSS group (Fig. 1D). Furthermore, the severity of colitis was histologically evaluated. Histological inflammation scores were significantly lower in the DSS + bortezomib group than in the DSS group (Fig. 2A and B). These findings indicate that bortezomib inhibited the development of DSS colitis.

### Bortezomib inhibits NF-κB activation in colonic epithelial cells

**Fig. 3:**
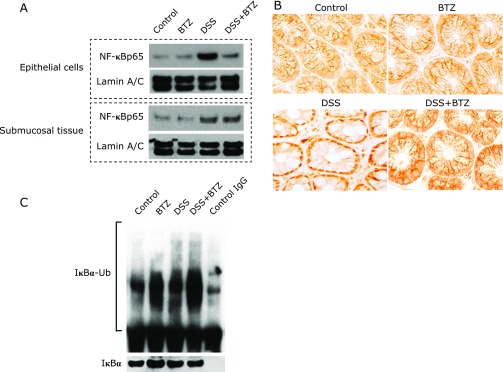
The effect of bortezomib on NF-κB activation. (A) Immunoblot for NF-κBp65 in the nuclear protein isolated from colonic epithelial cells and submucosal tissues in DSS-colitis mice. Representative picture of four independent experiments. (B) Immunohistochemical staining for NF-κBp65 in the colon sections (original magnification ×400). NF-κBp65 was detected in the nucleus of the epithelial cells in the DSS group, and this was blocked in the DSS plus bortezomib group. (C) Cytoplasmic protein extracted from isolated colonic epithelial cells were immunoprecipitated for IκBα and then analyzed by immunoblot using antibody against ubiquitin. Representative picture of 4 independent experiments. BTZ, bortezomib.

The effect of bortezomib on activation of NF-κB in colonic tissue was examined. Nuclear proteins were extracted from colonic epithelial cells of tissues and submucosal tissues, and immunoblotting was performed. In the study using colonic epithelial cells, the translocation of NF-κBp65 into the nucleus was markedly suppressed in the DSS + bortezomib group compared to the DSS group (Fig. 3A, upper panel), but in the submucosal tissue there was no difference between the same two groups (Fig. 3A, lower panel). In addition, the translocation of NF-κBp65 into the nucleus in colon tissue was examined using immunohistochemical staining. Similar to the findings of the immunoblotting, translocation of NF-κB into the nucleus in colonic epithelial cells was observed in the DSS group, and this was markedly suppressed in the DSS + bortezomib group (Fig. 3B). Furthermore, ubiquitinated IκBα in the cytoplasm of colon epithelial cells was examined using immunoprecipitation and immunoblot. There was an increase in the cytoplasmic ubiquitinated IκBα in the DSS + bortezomib group compared to the DSS group (Fig. 3C). These results indicate that bortezomib administration suppressed the activation of NF-κB through inhibition of degradation of ubiquitinated IκBα in colonic epithelial cells.

### Bortezomib directly inhibits the activation of NF-κB in colonic epithelial cells

**Fig. 4:**
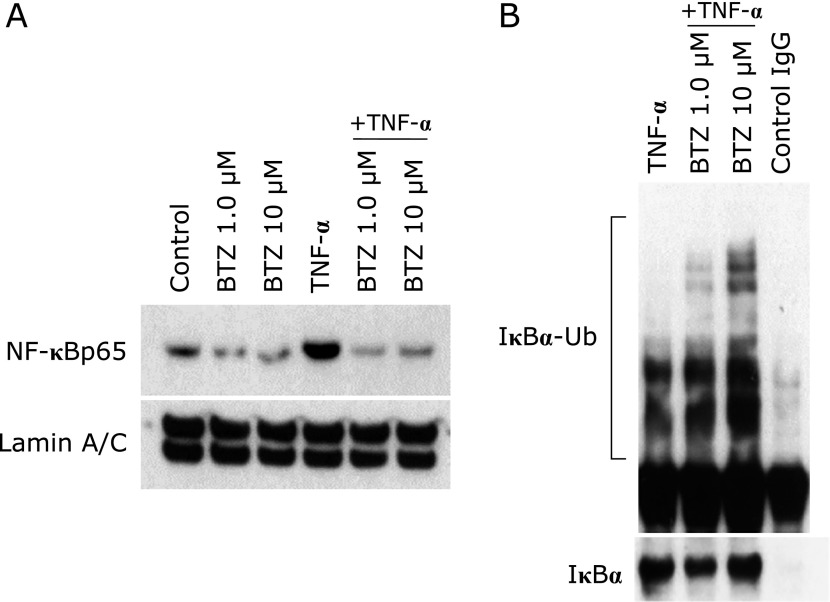
The effect of bortezomib on NF-κB activation *in vitro*. (A) Nuclear protein extracted from HT-29 cell line treated with bortezomib for 12 h and subjected to immunoblot. The translocation of NF-κB was suppressed by the administration of bortezomib. Lamin A/C was used as a loading control. The picture is representative of four independent experiments. (B) Cytoplasmic protein extracted from HT-29 cell line were immunoprecipitated for IκBα and then analyzed by immunoblot using antibody against ubiquitin. The picture is representative of four independent experiments. BTZ, bortezomib.

We evaluated whether suppression of NF-κB activation in colonic epithelial cells in the DSS + bortezomib group was due to direct action of bortezomib on the cells or was a secondary consequence of intestinal inflammation improvement. To do this we examined the effect of bortezomib on NF-κB activation *in vitro* using intestinal epithelial cell line HT-29 cells. Immunoblot using nuclear proteins extracted from HT-29 cells showed an induction of nuclear translocation of NF-κB by TNF-α (100 ng/ml), but bortezomib blocked this response (Fig. 4A).

Next, the accumulation of intracellular ubiquitinated IκBα was examined. HT-29 cells were stimulated with TNF-α (100 ng/ml) or TNF-α (100 ng/ml) + bortezomib (1.0 µM or 10 µM), and the expression of ubiquitinated IκBα in the cytoplasm was analyzed using immunoprecipitation and immunoblot. As shown in Fig. 4B, bortezomib dose-dependently increased ubiquitinated IκBα in the cytoplasm. These results indicate that bortezomib inhibited the nuclear translocation of NF-κB into the nucleus directly through blockade of ubiquitinated IκBα degradation in colonic epithelial cells.

### Effect of bortezomib on mRNA expression of inflammatory cytokines in colonic mucosa

**Fig. 5:**
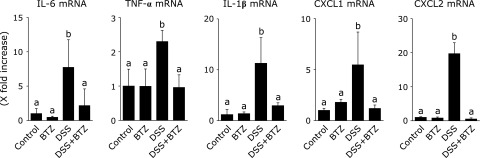
The effect of bortezomib on the expression of proinflammatory cytokines. Mucosal mRNA expression of proinflammatory cytokines. The expressed cytokine mRNA was converted to a value relative to β-actin mRNA and revealed as a relative increase to the results for control mice. The data are expressed as means ± SEM (*n* = 10 mice/group). Values not sharing a letter denote significant differences (*p*<0.05). BTZ, bortezomib.

The mRNA expression of inflammatory cytokines and chemokines in colonic epithelial cells was analyzed using real-time PCR. As shown in Fig. 5, mRNA expression of IL-6, TNF-α, IL-1β, CXCL1 and CXCL2 in colon epithelial cells was significantly reduced in the DSS + bortezomib group as compared to the DSS group. These results indicate that administration of bortezomib inhibited expression of inflammatory mediators in colonic epithelial cells.

### Effect of bortezomib on proliferation of colonic epithelial cells

**Fig. 6:**
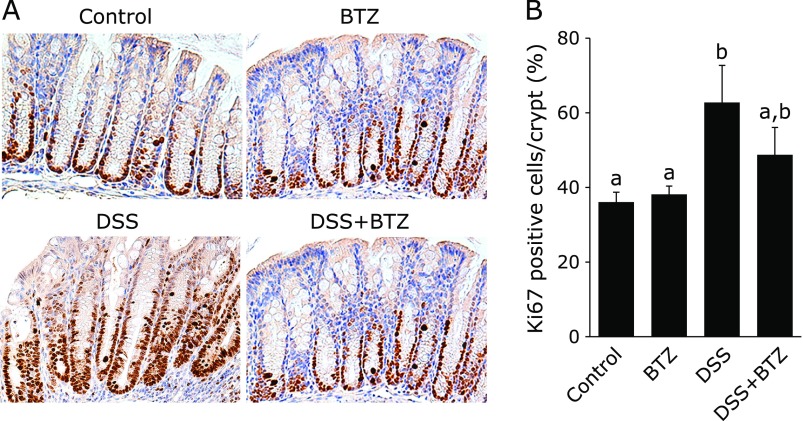
The effect of bortezomib on the proliferation of colonic epithelial cells. (A) Immunohistochemical staining for Ki67 in the colon sections (original magnification ×200). Ki67 was detected in the nucleus of the crypt. Representative picture of 4 independent experiments. (B) Percentage of Ki-67-positive cells per crypt. The data are expressed as means ± SEM (*n* = 10/group). Values not sharing a letter denote significant differences (*p*<0.05). BTZ, bortezomib.

Activation of NF-κB has been reported to be involved in proliferation of colonic epithelial cells. Epithelial cell proliferation was evaluated by immunohistochemical staining with Ki-67.^(22)^ In both the DSS group and the DSS + bortezomib group, a significant increase in the number of Ki-67 positive cells per crypt was observed as compared with the control group and the bortezomib group (Fig. 6A and B). There was no significant difference in the number of Ki-67 positive cells per crypt between the DSS group and the DSS + bortezomib group (Fig. 6A and B). Similarly, comparing the control group and the bortezomib group, there was no significant difference in the number of Ki-67 positive cells per crypt (Fig. 6A and B). These results indicate that growth of colon epithelial cells is not suppressed at the concentration of bortezomib used in this study.

## Discussion

In this study, we report that development of DSS-colitis was suppressed by bortezomib. As for its mechanism, bortezomib inhibited proteasome degradation of ubiquitinated IκBα in intestinal mucosa, and this was accompanied by suppression of NF-κB activation and mucosal expression of inflammatory cytokines and chemokines. These findings suggest that bortezomib could be one of the treatment options for IBD.

NF-κB is a transcription factor that regulates the expression of inflammatory cytokines, chemokines, anti-apoptotic molecules and growth factors, and has important functions such as regulation of immune and inflammatory responses, cell differentiation and cell proliferation.^(23,24)^ On the other hand, it has been reported that constitutive activation of NF-κB is related to inflammatory diseases such as IBD, rheumatoid arthritis and malignant tumors.^(11)^ Indeed, it is recognized that NF-κB activation is significantly higher in the inflamed mucosa of IBD patients compared to healthy individuals.^(25–27)^ It has also been shown that development of experimental colitis is blocked by suppression of NF-κB activation using antisense oligonucleotides.^(27,28)^ Furthermore, it has been reported that proteasomes isolated from IBD patients have a greater ability to activate NF-κB compared with healthy individuals.^(29)^ Thus, previous studies have suggested that NF-κB is a therapeutic target for many inflammatory and malignant disorders. At the present time, however, the only drugs clinically administered for the inhibition of NF-κB are proteasome inhibitors. Therefore, bortezomib is considered to be a treatment option for IBD.

Proteasome is known to play an important role in the regulation of NF-κB activation via degradation of ubiquitinated IκBα.^(8,9,14)^ Bortezomib inhibits the function of proteasome, thereby suppressing the activation of NF-κB. In this study, it was confirmed that nuclear translocation of NF-κBp65 in colonic epithelial cells during intestinal inflammation was markedly suppressed by bortezomib, and this was accompanied by inhibition of degradation of ubiquitinated IκBα in colon epithelial cells. Furthermore, *in vitro* studies using HT-29 cells showed that bortezomib has a direct effect on colonic epithelial cells. From these observations, it is suggested that the suppressive effect of bortezomib on DSS-colitis is closely associated with inhibition of proteasome degradation of ubiquitinated IκBα in the colonic epithelial cells and this leads to a suppression of NF-κB activation. The importance of the observation of epithelial NF-κB activation may be supported by the previous reports that mice overexpressing NF-κB specifically in the intestinal epithelium spontaneously develop colitis and exhibit increased susceptibility to DSS.^(30,31)^

There have been many studies published on the effect of bortezomib on multiple myeloma. In a study using a mouse model of multiple myeloma, bortezomib was used at a relatively higher dose (0.7–1.2 mg/kg) than the dose used in this study (0.35 mg/kg).^(32–34)^ It was suggested that bortezomib may need to be used at a high dose in order to exert its effect on bone marrow-derived cells including immune cells. This may be supported by our observation that inhibition of NF-κB activation by bortezomib was detected in colonic epithelial cells but not in immune cells that had infiltrated into the submucosa. Further investigation is necessary to identify why the effect of bortezomib expresses colonic epithelial cells as the main target.

In this study, bortezomib inhibited the expression of inflammatory cytokines (IL-6, TNF-α and IL-1β) and chemokines (CXCL-1 and CXCL-2) in colonic epithelial cells. These inflammatory cytokines and chemokines are increasingly expressed in IBD patients and are known to play an important role in the pathology of IBD.^(13)^ Furthermore, it has been reported that activation of NF-κB is involved in the regulation of expression of these inflammatory mediators.^(9,24)^ In this study, it is suggested that bortezomib inhibited the expression of inflammatory mediators by inhibiting activation of NF-κB in colon epithelial cells. In previous reports, the effects of bortezomib on experimental colitis have not been consistent. MG132, one of proteasome inhibitors, exacerbates DSS enteritis,^(35)^ and this is a contradictory observation to the findings of this study. It has been reported that the dose range that bortezomib can be safely used is relatively narrow. Considering the previous report, it has been shown that a low dose (0.35 mg/kg/day) of bortezomib inhibited DSS colitis, while a high dose (0.5–1.0 mg/kg/day) of bortezomib had no effects. Alternatively, administration of MG 132 has been shown to cause about 60% of mice to die or suffer from worsening colitis at the time of DSS colitis induction,^(36,37)^ and *in vitro* studies have reported that MG132 suppresses intestinal epithelial cell proliferation in a dose-dependent manner.^(35)^ In this study, it was shown that administration of bortezomib at a low dose (0.35 mg/kg/day) inhibits colitis and does not inhibit proliferation of colonic epithelial cells. Based on these findings, it is suggested that low doses of bortezomib inhibit colitis, but in high doses of bortezomib, excessive suppression of NF-κB disrupts the proliferation of intestinal epithelial cells and leads to aggravation of colitis. It is suggested that a dosage adjustment may be needed for the therapeutic use of bortezomib in IBD. Since the effect of bortezomib is known to be concentration-dependent, further investigation is considered to be necessary for examination of the concentration that exerts the most effect of suppressing colitis without causing adverse events.

In this study, we demonstrated that bortezomib, a protease inhibitor, inhibited the nuclear translocation of NF-κB in colonic epithelial cells by suppressing the degradation of IκBα and and contributed to an improvement in DSS colitis. Our study suggests that bortezomib may be a new treatment option for IBD.
