# Supplementary material for: Ameliorating effects of bortezomib, a proteasome inhibitor, on development of dextran sulfate sodium-induced murine colitis
Source: J Clin Biochem Nutr. 2018 Jun 8;63(3):217–23. doi: 10.3164/jcbn.18-42 (PMC6252295; doi:10.3164/jcbn.18-42)
Supplement: Supplemental Table 1 [file jcbn18-42st01.pdf]

Supplemental Table 1. Antibodies used in this study

Antibodies	Clone	Manufacturer
rabbit anti-NF-κBp65	C-20	Santa Cruz Biotechnology, Dallas, TX
mouse anti-IκBα	H-4	Santa Cruz Biotechnology, Dallas, TX
mouse anti-Lamin A/C	4C11	Cell Signaling Technology, Beverly, MA
rabbit anti-ubiquitin	ab7780	Abcam, Cambridge, MA
rabbit anti-Ki67	ab16667	Abcam, Cambridge, MA
mouse anti-NF-κBp65	12H11	EMD Millipore Corporation, Billerica, MA
HRP-labeled anti-mouse IgG		GE Healthcare UK Ltd., Chalfont, UK
HRP-labeled anti-rabbit IgG		GE Healthcare UK Ltd., Chalfont, UK
HRP-labeled anti-mouse IgG		Vector Laboratories, Burlingame, CA
