# Supplementary material for: Ameliorating effects of bortezomib, a proteasome inhibitor, on development of dextran sulfate sodium-induced murine colitis
Source: J Clin Biochem Nutr. 2018 Jun 8;63(3):217–23. doi: 10.3164/jcbn.18-42 (PMC6252295; doi:10.3164/jcbn.18-42)
Supplement: Supplemental Table 2 [file jcbn18-42st02.pdf]

Supplemental Table 2. PCR primers used in this study

Gene		Sequence 5'-3'
IL-1β	sense	CAGGATGAGGACATGAGCACC
	anti-sense	CTCTGCAGACTCAAACTCCAC
IL-6	sense	GACAAAGCCACACTCCTTCAGAGA
	anti-sense	CTAGGTTTGCCGATAGATCTC
TNF-α	sense	ATGAGCACAGAAAGCATGATC
	anti-sense	TACAGGCTTGTCACTCGAATT
CXCL1	sense	CCGAAGTCATAGCCACACTC
	anti-sense	CTCCGTTACTTGGGGACACC
CXCL2	sense	AGAAGTCATAGCCACTCTCAAG
	anti-sense	GCTCCTCCTTTCCAGGTCAG
β-actin	sense	GTGGGCCGCCCTAGGCACCA
	anti-sense	CGGTTGGCCTTAGGGTTCAGGGGGG
